# Promoter Polymorphism G-6A, which Modulates Angiotensinogen Gene Expression, Is Associated with Non-Familial Sick Sinus Syndrome

**DOI:** 10.1371/journal.pone.0029951

**Published:** 2012-01-05

**Authors:** Jan-Yow Chen, Ying-Ming Liou, Hong-Dar Isaac Wu, Kuo-Hung Lin, Kuan-Cheng Chang

**Affiliations:** 1 Department of Life Sciences, National Chung Hsing University, Taichung, Taiwan; 2 Division of Cardiology, Department of Medicine, China Medical University Hospital, Taichung, Taiwan; 3 School of Medicine, China Medical University, Taichung, Taiwan; 4 Graduate Institute of Basic Medical Science, China Medical University, Taichung, Taiwan; 5 Department of Applied Mathematics and Institute of Statistics, National Chung Hsing University, Taichung, Taiwan; Universidad Europea de Madrid, Spain

## Abstract

**Background:**

It is well known that familial sick sinus syndrome (SSS) is caused by functional alterations of ion channels and gap junction. Limited information is available on the mechanism of age-related non-familial SSS. Although evidence shows a close link between arrhythmia and the renin-angiotensin system (RAS), it remains to be determined whether the RAS is involved in the pathogenesis of non-familial SSS.

**Methods:**

In this study, 113 patients with documented non-familial SSS and 125 controls were screened for angiotensinogen (AGT) and gap junction protein-connexin 40 (Cx40) promoter polymorphisms by gene sequencing, followed by an association study. A luciferase assay was used to determine the transcriptional activity of the promoter polymorphism. The interaction between nuclear factors and the promoter polymorphism was characterized by an electrophoretic mobility shift assay (EMSA).

**Results:**

Association study showed the Cx40 -44/+71 polymorphisms are not associated with non-familial SSS; however, it indicated that four polymorphic sites at positions -6, -20, -152, and -217 in the AGT promoter are linked to non-familial SSS. Compared to controls, SSS patients had a lower frequency of the G-6A AA genotype (OR 2.88, 95% CI 1.58–5.22, *P* = 0.001) and a higher frequency of the G allele at -6 position (OR 2.65, 95% CI 1.54–4.57, *P* = 0.0003). EMSA and luciferase assays confirmed that nucleotide G at position -6 modulates the binding affinity with nuclear factors and yields a lower transcriptional activity than nucleotide A (*P*<0.01).

**Conclusion:**

G-6A polymorphism, which modulates the transcriptional activity of the AGT promoter, may contribute to non-familial SSS susceptibility.

## Introduction

Sick sinus syndrome (SSS), including profound sinus bradycardia, sinus arrest, sino-atrial exit block, and tachy-bradycardia, is a group of abnormal heart rhythms presumably caused by a malfunction of the sinus node [1], [2]. The syndrome is prevalent in 1 out of every 600 individuals over the age of 65 years, and accounts for approximately 50% of pacemaker implantations [1], [2]. Growing evidence has shown that genetic mutations in the hyperpolarization-activated cyclic nucleotide-gated cation channel (HCN-4), the cardiac sodium channel (SCN5A), and gap junction protein (connexin) may lead to familial SSS [3]–[5]. In contrast to the progress in illustrating the mechanism for familial SSS, limited information is available regarding the mechanism of age-related non-familial SSS [6], [7].

Gap junctions composed of connexin (Cx) molecules are responsible for the electrical coupling of cardiac myocytes. In the human heart, there are 3 cardiac connexin isotypes, Cx40, Cx 43 and Cx45. Cx40 is the major isotype expressed in the atrium. In Cx40 knockout mice, increased atrial vulnerability has been shown to cause arrhythmogenesis [8]. In addition, Cx40 promoter polymorphism has been linked to congenital atrial standstill and atrial arrhythmia [4], [9]. However, it is still unclear whether alterations in gap junction proteins would contribute to non-familial SSS.

It is generally accepted that the renin-angiotensin system (RAS) can modulate the functions of the sinus node and cardiac conduction system [10]–[12]. Angiotensin II is known to induce cardiac fibrosis and myocardial hypertrophy through angiotensin II type I (AT1) receptors [13]. A study using transgenic mice demonstrated that overexpression of the AT1 receptor in the myocardium, which was lethal, was associated with myocyte hyperplasia, heart block, and sinus bradycardia [14]. However, the role of RAS in the pathogenesis of age-related non-familial SSS remains to be determined.

Studies analyzing the association between angiotensinogen (AGT) promoter polymorphism and stroke have indicated that polymorphic alterations of the AGT promoter modulate its transcriptional activity and cause cerebral vascular diseases [15], [16]. In addition, a study using AGT knockout mice showed a feedback mechanism for regulating the expression of RAS molecules and the AT1 receptor [17]. Thus, we hypothesized that AGT promoter polymorphism modulates the expression of RAS molecules and thereby influences sinus node function. Here, the data reported indicates a possible relationship between age-related non-familial SSS and the AGT promoter polymorphism. Apparently, polymorphic variations in the AGT promoter might contribute to non-familial SSS susceptibility by modulating the activity of RAS in patients.

## Methods

### Ethics statement

The study protocol was approved by the Institutional Review Board of China Medical University Hospital. Written informed consent was obtained from each participant or each participant's guardian after a full explanation of the study. The investigation conformed with the principles outlined in the Declaration of Helsinki.

### Study population

A total of 113 consecutively eligible patients with documented SSS were studied. SSS was diagnosed by symptomatic bradycardia with evidence of sinus node dysfunction. The criteria for inclusion were symptomatic bradycardia with a documented sinus pause of greater than 3 seconds or sinus bradycardia of less than 40 beats/min for more than 1 min while awake [18], [19]. Other supporting evidences were provided by a cardiac electrophysiologic study to determine the prolonged corrected sinus nodal recovery time. Long sinus pauses and profound sinus bradycardia were also examined by using a series of electrocardiograms (ECG) and ambulatory ECG. All SSS patients met the indications for permanent pacemaker implantation. Patients with a history of familial SSS, severe systemic disease, acute coronary syndrome, neurogenic or drug-induced bradycardia, or bradycardia with reversible cause were excluded from this study. The control group consisted of 125 age- and sex-matched unrelated volunteer patients who were free of SSS and underwent clinical follow-up in the cardiovascular outpatient department of the same hospital.

### Genotyping and association study

Blood samples from patients were prepared, and genomic DNA was isolated using a DNA extraction kit (Illustra^TM^, GE Healthcare). Polymerase chain reactions (PCRs) were performed with 100 ng genomic DNA, 2–6 pmol of selected primers, 1X Taq polymerase buffer, and 0.25 units of AmpliTaq Gold^TM^ polymerase (Roche) in a final reaction volume of 50 µL using a programmable thermal cycle (GeneAmp PCR system 2700, Applied Biosystems, CA). The primers for the AGT promoter were 5′-CCTCTTGGGGGTACATCTCC-3′ (forward) and 5′-TCCTAGCCCACAGCTCAGTT-3′ (reverse). The primers for the Cx40 promoter were 5′-AGGCTACGAGGAGGTGGA-3′ (forward) and 5′-AACTCACAGGTAGAAAGAAAGAGC-3′ (reverse). The gene sequences of the PCR products were subsequently determined by using a gene sequencing analyzer (ABI 3730 XL DNA Analyzer, Applied Biosystems). An association study between gene promoter polymorphisms and SSS was performed to measure the frequency of the genotypes and alleles of the Cx40 and AGT promoters in SSS and control groups.

### Construction of expression vectors, transfection, and luciferase activity measurement

**Figure 1:**
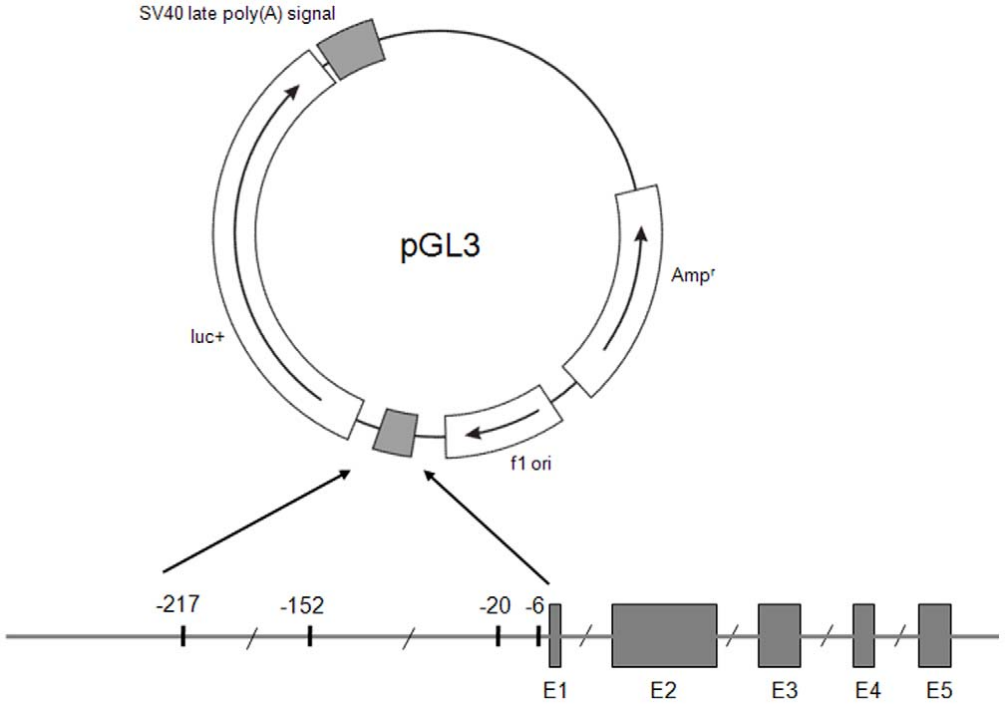
Construction of expression variants in pGL3 vector using oligonucleotides for the proximal AGT promoter. The oligonucleotides containing the proximal promoter region of the AGT gene from position −290 to +35 were ligated in the pGL3 vector to produce the reporter construct. *luc*+  =  cDNA encoding the modified firefly luciferase; Amp^r^  =  gene conferring ampicillin resistance; f1 ori  =  origin of replication derived from filamentous phage; E  =  exon.

The association study showed that polymorphic sites in the AGT promoter were located within the proximal region of the promoter. Therefore, this region (position −290 to +35 relative to transcription starting site) was amplified by PCR from the genomic DNA of homozygous patients. Primers to amplify AGT polymorphisms were designed to contain the restriction sites of *Mlu*I and *Bgl*II (for cloning) and polymorphic sites for AGT promoter: forward primer, 5′- ACCGACGCGTAGATGCTCCCGTTTCTGG -3′ (artificial *Mlu*I restriction site underlined); reverse primer, 5′-CGGAAGATCTTCTGCTGTAGTACCCA-3′ (artificial *Bgl*II restriction site underlined) (Figure 1). After digestion with *Mlu*I and *Bgl*II, PCR products were ligated into the corresponding restriction sites of the pGL3 plasmid containing the luciferase reporter gene according to the manufacturer's instructions (Promega). The promoter-luciferase constructs containing the -6G and -6A polymorphic sites are defined as P(-6G) and P(-6A), respectively.

The constructs were transiently transfected into HepG2 (cell line-derived from human hepatoma; HB-8065; ATCC) and cultured in Dulbecco's Modified Eagle Medium (DMEM) without serum using a transient liposome (Lipofetamine2000; Invitrogen, Carlsbad, CA) cotransfection method. A control vector containing the beta-galactosidase gene (Promega; 0.2 µg) was used as an internal control of transfection. Luciferase assays were performed using the Dual-Light Luciferase Assay System (PerkinElmer).

### Electrophoretic mobility shift assay

Electrophoretic mobility shift assays (EMSA) were performed by using the EMSA “Gel Shift” Kit (Panomics, Fremont, CA, USA). To determine the essential role of the specific position at -6 at the proximal segment of the AGT promoter, 2 different lengths of oligonucleotides containing the nucleotide A or G at -6 of the AGT promoter were designed for the longer oligonucleotide, G33 or A33, and for the shorter oligonucleotide, G23 or A23. The sequences for these double-stranded oligonucleotides are listed below with the polymorphic sites underlined:

G23:5′-GTGACCCGGCCGGGGGAAGAAGC-3′, A23:5′-GTGACCCGGCCAGGGGAAGAAGC-3′, G33:5′-AAATAGGGCATCGTGACCCGGCCGGGGGAAGAA-3′, A33:5′-AAATAGGGCATCGTGACCCGGCCAGGGGAAGAA-3′.

These synthesized oligonucleotides were labeled with biotin at the 3′ end. The biotinylated oligonucleotide (10 ng/µL) was added to nuclear extracts of HepG2 cells after their nuclear proteins were incubated with poly (dI-dC) (1 µg/µL) and binding buffer for 5 minutes. The specific binding was evidenced by adding a 50- to 100-fold excess of corresponding non-labeled oligonucleotide with nuclear extracts prior to the addition of the biotinylated probe for each sample preparation. Binding reaction mixtures were then incubated for 30 min at 15°C. After electrophoresis, gels were transferred to nylon membranes. For detection of bound oligonucleotides, membranes were blocked using blocking buffers (Panomics EMSA Gel-Shift Kit) followed by the addition of Streptavidin-HRP, and blots were developed by ECL according to the manufacturer's instructions.

### Resting ECG recordings

The patients resting in supine position for 10 minutes were performed ECG recordings. Both heart rate and PR interval were taken into account as essential factors affecting sinus node function and rhythmic conduction in the heart [1]. The control patients without SSS were divided into two groups with age and sex match according to AGT genotype. Six patients with chronic atrial fibrillation or without ability to maintain sinus rhythm during ECG recording were excluded.

### Holter ECG

A Holter ECG monitor is a portable recorder that can continuously record the heart rate and rhythm for at least 24 hours [20]. In the present study, Holter ECG monitoring was performed for 24 hours for those patients who required monitoring for a longer period to evaluate their heart rhythm or to provide supporting evidence for the diagnosis of rhythm disorders.

### Electrophysiologic study

An electrophysiologic study is an invasive tool that can evaluate sinus node function and the conduction system [21]. An electrophysiologic study can provide supporting evidence for the diagnosis of SSS or further evaluation of bradycardia. Some patients with non-familial SSS can be diagnosed by series ECG, bedside ECG mornitoring, ECG telemetry monitoring and concurrent symptoms and/or signs of bradycardia [1], [18], [19]. Therefore, an electrophysiologic study was not performed for part of patients with non-familial SSS. All patients in the control group are free of symptoms and signs of non-familial SSS based on careful history taking, resting ECG and heart rate follow-up. Therefore, an invasive electrophysiologic study was not performed for the control group due to ethic issue. The invasive electrophysiologic study was performed when bradycardia occurred incidentally and could not be well evaluated by non-invasive methods or when a serious underlying mechanism for bradycardia was suspected [21]. The phenomenon of post-pacing depression of the sinus node was utilized to evaluate the sinus node function for the study subjects by analyzing the sinus node recovery time (SRT) as described in a previous report [22]. The SRT is defined as the interval between the last paced P wave and the following sinus P wave. The corrected SRT (CSRT) is defined as the recovery interval in excess of the sinus cycle (SRT -sinus cycle length). The SRT was measured following overdrive suppression for sinus node by right atrial pacing at rates of 100 to 150 beats/min for 2 minutes at each pacing rate. A CSRT longer than 525 ms is considered prolonged [22].

### Statistical analysis

Student's *t* test was used when the continuous data were normally distributed; otherwise, the nonparametric Mann-Whitney *U* test was used. Categorical data were compared by the conventional chi-square test if the observation numbers in all categories were larger than 5; otherwise, the Fisher exact test was used. Numeric variables for the promoter genotypes were compared using one-way analysis of variance (ANOVA). For each polymorphism, the genotype proportions with Hardy–Weinberg equilibrium (HWE) were assessed by using the conventional χ^2^ goodness-of-fit test. Haplotype profile analysis for the polymorphisms was estimated by using Haploview software [23]. Owing to short distances between each polymorphism location on the AGT gene, polymorphisms probably did not separate by recombination and had linkage disequilibrium (LD) [24]. Thus, pairwise measurement of LD was performed to test the LD between the polymorphisms. D' was used to estimate LD. Because the magnitude of D' strongly depends on the sample size, and it is known to increase when a small number of samples or rare alleles are examined, we also utilized the r^2^ value to confirm LD. Expectation-maximization (EM) based haplotype frequency estimation with a permutation test was performed to determine whether any specific haplotypes are associated with SSS on the basis of previous reports [24]–[26]. Statistical significance of LD was defined as r^2^>1/3 and D′>0.7, as suggested by previous reports [26]–[27]. A *P*-value <0.05 was considered to be statistically significant.

## Results

### Patient characteristics

The clinical features of SSS patients and controls are summarized in Table 1. There were no significant differences in age, gender, percentage of patients with hypertension, diabetes mellitus (DM), coronary artery disease (CAD), atrial fibrillation (AF), and left ventricle dysfunction between groups.

**Table 1:** General characteristics of patients included in the study.

	SSS	Control	
	(N = 113)	(N = 125)	
**Age (years)**	68.9±10.7	69.0.5±8.7	0.493^**^
**Gender (male/female)**	37/76	46/79	0.586^§^
**BW (kg)**	60.6±9.88	62.2±9.0	0.181*
**Height (cm)**	157.8±7.9	158.2±7.9	0.761^**^
**HT (n, %)**	41 (36.3%)	55 (44.0%)	0.226 ^§^
**DM (n, %)**	28 (24.8%)	23 (18.4%)	0.231 ^§^
**CAD (n, %)**	12 (10.6%)	15 (12.0%)	0.737 ^§^
**AF (n, %)**	23 (20.4%)	22 (17.6%)	0.622 ^§^
**LAD (mm)**	36.3±6.2	36.1±6.5	0.627^**^
**LVIDd (mm)**	49.0±5.4	48.6±6.67	0.472^**^
**LVEF (%)**	71.4±10.7	71.3±12.7	0.616*

*Student t test; **Mann-Whitney U test; ^§^χ^2^ test; SSS  =  sick sinus syndrome; HT  =  hypertension; DM  =  diabetes mellitus; CAD  =  coronary artery disease; LAD  =  left atrial dimmesion; LVIDd  =  left ventricular end diastolic dimension; LVEF  =  left ventriclar ejection fraction.

### HWE tests and LD measurements

**Figure 2:**
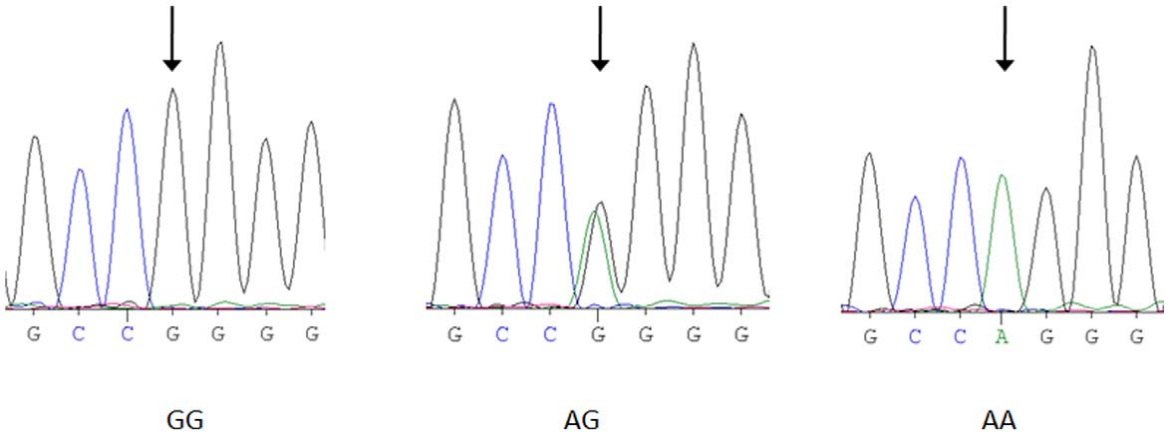
AGT G-6A polymorphism genotyping by direct sequencing. The arrows indicate the polymorphic site of GG, AA and AG genotypes.

Four polymorphic sites were found at positions -6, -20, -152, and -217 within the promoter region of the AGT gene (Figure 2 and Figure S1, S2, and S3). The HWE genotype distributions were assessed for each AGT promoter polymorphism G-6A, A-20C, G-152A, and G-217A by the conventional chi-squared goodness-of-fit test. The *P*-value was 0.33, 0.65, 0.47, and 0.29, respectively. In addition, the same test was also performed for the SSS and control groups separately. The AGT genotype distribution in the SSS and control groups did not significantly deviate from the HWE (*P*>0.2 in each polymorphism).

**Figure 3:**
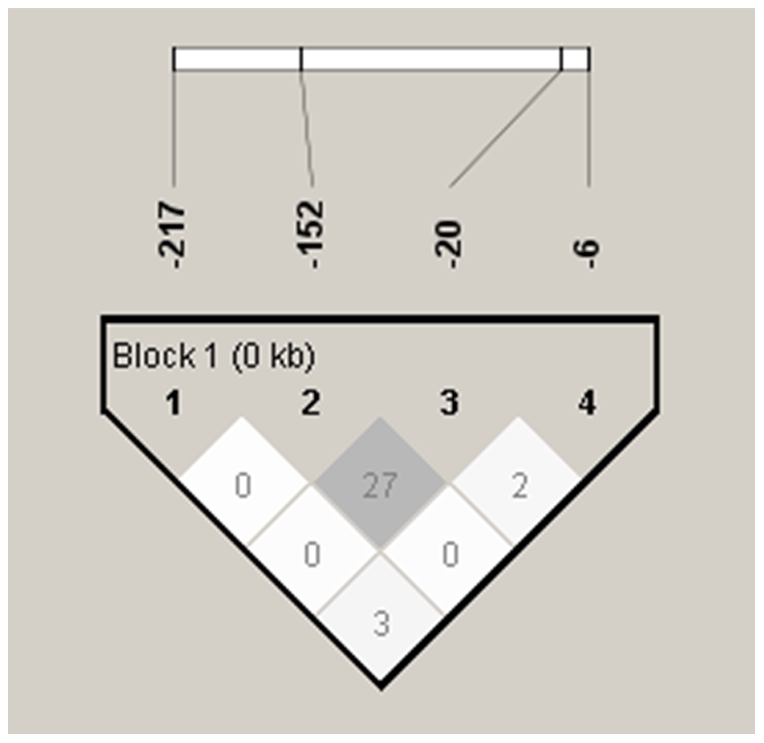
Linkage disequilibrium plot of AGT promoter polymorphisms. Pairwise linkage disequilibrium analysis shows r^2^ (×100) values. The intensity of gray is proportional to r^2^.

The pairwise linkage among these four polymorphic sites on the AGT promoter gene was evaluated by the LD test using D' and r^2^. The D' values of the loci pairs for -6/-20, -6/-152, -6/-217, -20/-152, -20/-217, and -152/-217 were 1, 1, 1, 1, 0.463, and 0.699, respectively. The corresponding values for r^2^ were 0.028, 0.008, 0.039, 0.277, 0.005, and 0.001, respectively (Figure 3). The D' values indicated a significant linkage in the loci pairs of -6/-20, -6/-152, -6/-217 and -20/-152. However, the r^2^ values for these three loci pairs were low. This inconsistency between the D' and r^2^ values may be due to the small sample size in this study [26]. The high D' values and the low r^2^ values of these 4 loci pairs (-6/-20,-6/-152, -6/-217 and -20/-152) suggest an incomplete linkage among these 4 loci pairs, which explains the wide range of haplotypes for the AGT gene.

Two polymorphic sites were found at position of -44 and +71 in Cx40 gene (Figure S4). The Cx40 genotype distribution in total population, SSS patients and control groups did not significantly deviate from the HWE (*P = *0.46, 0.38 and 0.65, respectively). These two Cx40 polymorphisms were in complete linkage disequilibrium. The patients with allele G at position -44, consistently had A at -71 position and vice versa.

### Relationship between the AGT promoter haplotypes and SSS

In the present study, five major haplotypes in the AGT promoter showing a frequency of >0.01 were identified, and their relationship with SSS was examined. The GGAG haplotype (-217G, -152G, -20A, -6G) occurred with a significantly higher frequency in the SSS group compared to the control group (haplotype frequency: 0.2035 vs. 0.0880; OR = 2.65, *P* = 0.0003; Table 2).

**Table 2:** Haplotype frequency estimates of AGT gene in patients with sick sinus syndrome and controls.

Haplotype								
				Overall	SSS	Controls		
-217	-152	-20	-6	(N = 238)	(N = 113)	(N = 125)	OR	P
G	G	A	A	0.536	0.5097	0.5600	0.82	0.2719
G	G	A	G	0.143	0.2035	0.0880	2.65	0.0003
G	G	C	A	0.090	0.0686	0.1096	0.60	0.1158
A	G	A	A	0.178	0.1540	0.2000	0.73	0.1905
G	A	C	A	0.042	0.0504	0.0344	1.49	0.3859

*There results were confirmed by permutation test which also revealed that GGAG is the only significant candidate haplotype (P = 0.0007). Haplotypes are not listed if all the estimated frequencies are <0.01 in patients with sick sinus syndrome, controls, and overall population

### Single locus analysis of AGT promoter polymorphisms and SSS association

A significant difference was observed in the distribution of the genotypes at position -6 between SSS and control subjects (*P* = 0.001). The AA genotype frequency of G-6A was significantly lower in the SSS group than in the control group (OR = 2.88, 95% CI: 1.58–5.22, *P* = 0.001). The G allele frequency of G-6A was significantly higher in the SSS group than in the control group (20.4% vs. 8.8%, OR = 2.65, 95% CI: 1.54–4.57, *P* = 0.0003) (Table 3). Results of the haplotype analysis and single locus analysis indicate a significant association between G-6A polymorphism and SSS.

**Table 3:** Distribution of genotypes and alleles of Cx40 and AGT in patients with and without sick sinus syndrome.

Gene polymorphism	Genotypes and Alleles	SSS patients	Control patients	P
		(N = 113)	(N = 125)	
**Cx40 gene**				
G-44A	AA	10	14	
	AG	52	59	
	GG	51	52	0.77
	A:G	31.9%:68.1%	34.2%:65.8%	0.50
**AGT gene**				
G-6A	AA	70	103	
	AG	40	22	
	GG	3	0	0.001
	A:G	79.6% : 20.4%	91.2% : 8.8%	0.0003
A-20C	AA	85	89	
	AC	26	34	
	CC	2	2	0.83
	A:C	86.7% : 13.3%	84.8% : 15.2%	0.55
G-152A	AA	0	0	
	AG	12	9	
	GG	101	116	0.37
	A:G	5.3% : 94.7%	3.6% : 96.4%	0.36
G-217A	AA	3	3	
	AG	32	46	
	GG	78	76	0.39
	A:G	16.8% : 83.2%	20.8% : 79.2%	0.27

SSS  =  sick sinus syndrome. P values obtained based on χ^2^ test or Fisher's exact test; the upper P value is for comparison of genotype frequencies, and the lower is for allele frequencies.

### Genotypes and alleles distribution of Cx40 polymorphisms in SSS patients and controls

Owing to the Cx40 polymorphisms at positions -44 and +71 were in complete linkage disequilibrium, we only reported the results for Cx40 -44(GA) polymorphism. There was no significant difference in the distribution of allele frequency of -44G and -44A and genotype distribution of -44AA, -44AG and -44GG between SSS and control patients (P = 0.50 and 0.77, respectively). These results indicate no association between Cx40 -44 polymorphism and non-familial SSS (Table 3).

### Transcriptional activity of the various AGT promoter polymorphisms

**Figure 4:**
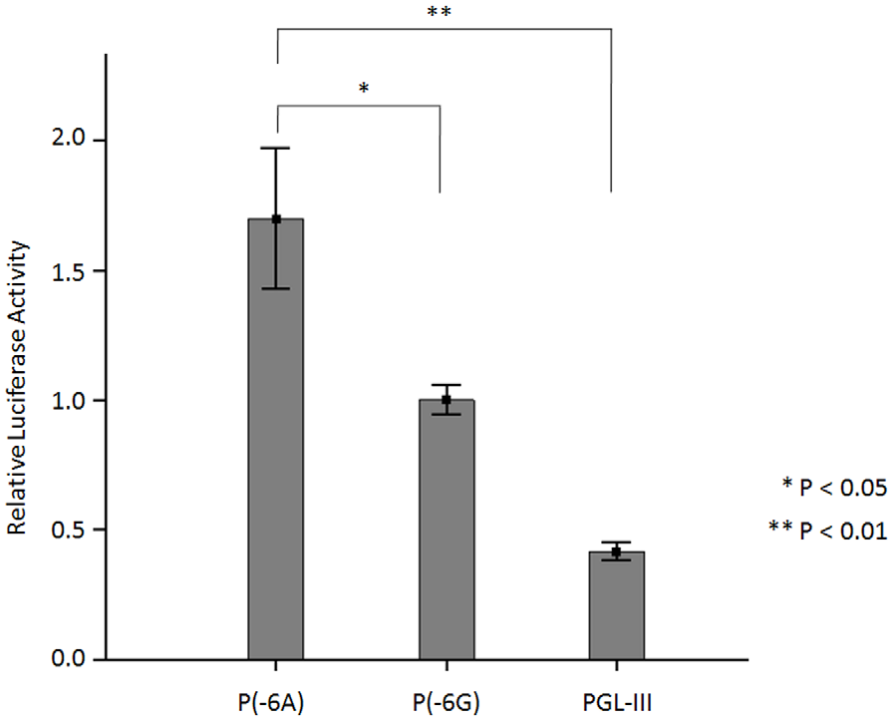
Comparison of the transcriptional activities of reporter constructs containing AGT proximal promoter polymorphisms in HepG2 cells. Transcriptional activities are presented as a ratio of the activity of the p(-6G) promoter-luciferase construct. pGL3 represents the blank vector, which does not contain any AGT promoter sequence. Values are presented as means±SE.

The effect of polymorphisms on AGT promoter activity was determined by transiently transfecting plasmids containing AGT promoter polymorphisms upstream of the luciferase gene into a hepatocyte (HepG2) cell line and measuring luciferase activity. The AGT promoter containing G at -6 (p(-6G)) had a lower transcriptional activity than the AGT promoter containing A at -6 (p(-6A)) in HepG2 cells ( P<0.05) (Fig. 4). This suggests that decreased AGT promoter activity in RAS is involved in the pathogenesis of SSS.

In the transcription activity study using the luciferase assay, we found that the AGT promoter containing G at -6 had a lower transcriptional activity than the AGT promoter containing A at -6. According to the transcriptional activity study by luciferase assay, we identified the homozygous AA genotype as the genotype with the highest transcription activity, the heterozygous AG genotype as the genotype with an intermediate transcriptional activity and the homozygous GG genotype as the genotype with the lowest transcription activity. Accordingly, the control group had a higher frequency of genotype with the highest transcription activity than the SSS group (control group vs. SSS group  = 103/125 (82.4%) vs. 70/113(61.0%), P<0.001). In contrast to the control group, the SSS-group possessed a higher frequency of genotypes with intermediate or the lowest transcriptional activity (SSS group vs. control group  = 43/113 (38.1%) vs. 22/125 (17.6%), p<0.001). These results indicate the G-6A polymorphism modulates the transcriptional activity of the AGT promoter and may contribute to non-familial SSS susceptibility.

### EMSA

**Figure 5:**
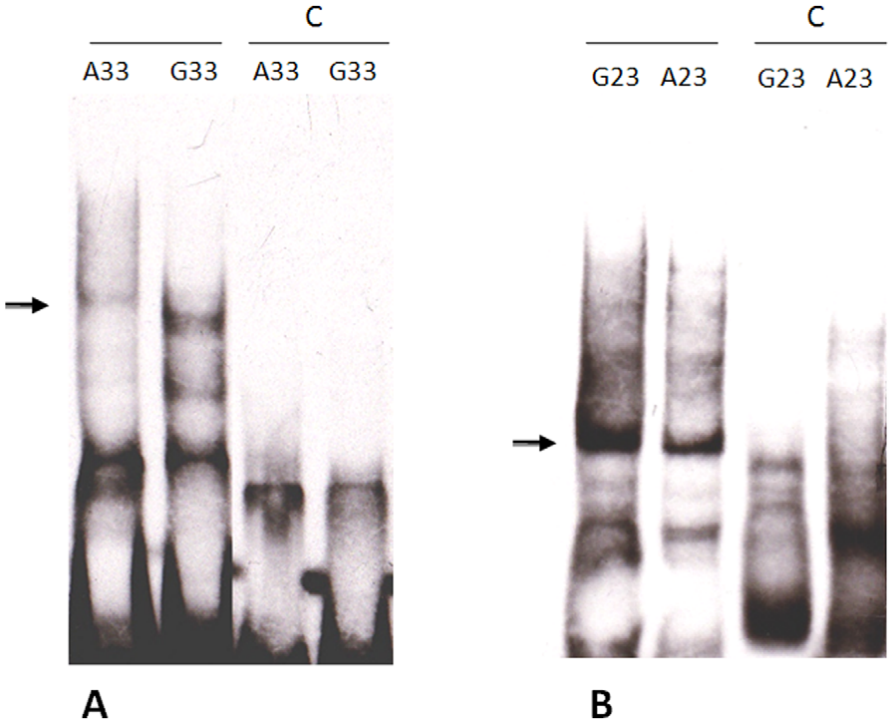
EMSA results of comparison of the binding affinities of biotinylated oligonucleotides. (A) Lanes 1 and 2 indicating the mobilities of labeled, biotinylated oligonucleotides (A33 and G33) with nuclear extracts. A stronger shift was observed in the G33 in comparison to the A33 oligonucleotide. Lanes 3 and 4 showing the competition experiments. The unlabeled oligonucleotides completely inhibited the specific binding complex of biotin-labeled A33 and G33 probes to nuclear extract. (B) Lanes 1 and 2 indicating the mobilities of labeled oligonucleotides (G23 and A23) with nuclear extracts. A stronger binding complex was observed in G23 in comparison with the A23 oligonucleotide. The unlabeled oligonucleotides completely inhibited the specific binding complex of biotin-labeled G23 and A23 probes to nuclear extract in lane 3 and 4. The arrow points to the specific nuclear complex, which binds with the labeled, biotinylated oligonucleotides. C  =  competitor.

The luciferase assay showing that the AGT transcription rate is modulated by the nucleotide substitution at position -6 of the proximal promoter reflects that polymorphic substitution of nucleotide -6 may alter the interaction between transcriptional factors and the proximal region of the AGT promoter, subsequently altering the transcription rate. To test this possibility, the direct binding experiment of EMSA was conducted to compare the formation of retarded complexes of oligonucleotide G33 or A33 with nuclear extracts from HepG2 cells. The results that were obtained showed that a stronger blot shift by nuclear proteins was observed for the biotin-labeled oligonucleotide G33 than for oligonucleotide A33. This retarded complex could be abolished by pretreatment of each sample preparation with each unlabeled corresponding oligonucleotide. Similarly, by adding a shorter biotin-labeled oligonucleotide (G23 or A23), a stronger blot shift by nuclear proteins was also observed for G23 than for A23 (Figure 5).

### Effects of AGT G-6A and Cx40 -44/+71 polymorphisms on heart rate and PR interval in control patients without SSS

To verify the functional association of G-6A polymorphism to non-familial SSS, we examined whether ATG G-6A polymorphism in control patients without SSS would affect their sinus node function. Both heart rate and PR interval were considered as critical factors in association with sinus node function and rhythmic conduction of the heart. The resting heart rate in the subjects with AG genotype is significant lower than the subjects with AA genotype (AG vs. AA = 68.2±12.2 vs. 74.4±11.9 beats/min, P = 0.03) (Table 4). This suggests that AGT G-6A polymorphism might play a role in sinus rate control. In addition, the subjects with AG genotype also showed a trend of longer PR interval than the subjects with AA genotype (AG vs, AA = 164.9±26.5 vs. 154.1±18.2 ms, P = 0.07) (Table 4). Consistently, the control patients with AG genotype have longer electrical conduction for the atria to ventricles that might lead to impaired atrioventricular conduction. Thus, these results suggested that the AGT G-6A polymorphism not only associates with non-familial SSS, but also has effects on the sinus node function in the control subjects. In contrast to the effect of AGT G-6A polymorphism on sinus node function, there were no significant differences in the resting heart rate among the subjects with different genotypes of the Cx40 -44/+71 polymorphism (AA (n = 14): AG (n = 55): GG (n = 50)  = 71.3±18.2 : 73.7±11.2: 73.3±11.4 beats/min, P = 0.70). There were also no significant differences in the PR intervals among the patients with the different genotypes of the Cx 40 -44/+71 polymorphism (AA: AG: GG = 156.8±17.2: 157.39±28.5: 154.5±23.2 ms, P = 0.38). These results indicate that the Cx40 -44/+71 polymorphisms had no effect on the sinus node function in the control subjects.

**Table 4:** Effects of AGT G-6A polymorphism on heart rate and PR interval measured by ECG recordings in control patients without SSS.

	Genotypes		
	AG	AA	P
	(N = 22)	(N = 97)	
Heart rate (beats/min)	68.2±12.2	74.4±11.9	0.03
PR interval (ms)	164.9±26.5	154.1±18.2	0.07

In additional to providing supporting evidence for diagnosis of non-familial SSS, 30 subjects of the control group were evaluated by Holter ECG monitoring for their heart rhythms. In the 30 subjects, the results showed a significant trend of a lower average heart rate with the AG genotype (n  = 10) than the AA genotype (n = 20) (AG vs. AA = 69.7±7.0 vs. 76.1±11.9 beats/min, p = 0.129). Although the results did not reach a statistically significant status, they are consistent with the result of sinus rate comparison by resting ECG in the control group. These results support the findings that the AGT G-6A polymorphism affects sinus node function in the control subjects. In contrast to the effect of G-6A polymorphism on average heart rate found by Holter ECG, there were no significant differences in the average heart rate among the subjects with the different genotypes of the Cx40 -44/+71 polymorphism (AA (n = 14): AG(n = 55): GG(n = 50)  = 71.0±12.4 : 75.7±11.7: 72.6±9.6 beats/min, P = 0.66). These results indicate that the Cx40 -44/+71 polymorphisms have no effect on the sinus node function in the control subjects.

### Electrophysiologic study and the genotypes of AGT G-6A polymorphism

An electrophysiologic study has been performed for 55 non-familial SSS patients (35 patients with the AA genotype, 18 patients with the AG genotype and 2 patients with the GG genotype). The CSRT was recorded in these 55 patients. There were no significant differences in CSRT among the AGT G-6A AA, AG and GG genotypes of these SSS patients (AA: AG: GG = 1425.97±825.27: 1573.17±1599.22: 1536.00±1005.51 ms, P = 0.83). The results indicated that electrophysiologic criteria for supporting SSS diagnosis were similar among the patients with AA, AG and GG genotypes. Although the SSS group showed a increased incidence of GG and AG genotypes compared with the control group (SSS group vs. control group = 43/113 (38.1%) vs. 22/125 (17.6%), p<0.001), the severity for supporting SSS diagnosis in AG and GG genotypes by CSRT was no less than that for the AA genotype.

## Discussion

It is well-known that malfunctions in HCN-4, SCN5A, and connexin are genetically associated with familial SSS. In addition, sinus node fibrosis has been reported to cause abnormal sinus node function [1]. Moreover, the risk for fibrosis-related SSS without genetic inheritance (non-familial SSS) is known to increase with age [1], [2]. Only a few studies have analyzed the influence of genetic characteristics on the development of fibrosis and age-related SSS. Since the pathogenesis of non-familial SSS is quite different from that of familial SSS, it is of interest to explore the underlying mechanism of non-familial SSS. In the present study, we showed that AGT promoter polymorphism is highly associated with non-familial SSS, possibly by modulating AGT expression. This result may provide useful information in the prevention of age-related non-familial SSS.

RAS has been implicated in cardiac fibrosis and sinus node dysfunction [13], [14]. It has been reported that angiotensin II mediates the proliferation of fibroblasts through the mitogen-activated protein kinase signaling pathway [28], [29]. Immunohistochemical studies with monoclonal antibody against the endogenous proteins of AGT in the heart visualized their expression in the cardiac conduction system [11]. Autoradiography also showed that angiotensin II binding sites (AT1 receptors) are localized in the sinus and atrioventricular nodes of rat hearts [12]. In addition, angiotensin II induces cardiac apoptosis via AT1 receptors in the conducting system [29]. These studies suggest that fibrosis-related non-familial SSS may be closely associated with alterations in RAS.

### Single locus, haplotype analyses, and transcriptional activity of AGT gene promoter polymorphisms

Although the present study showed four polymorphic sites at positions -6, -20, -152, and -217 in the promoter region of the AGT gene (Table 3), only the position at -6 with nucleotide substitution GA was found to be significantly different between control and SSS groups. In the haplotype analysis, we found 5 major haplotypes of the AGT promoter polymorphism in control and SSS patients (Table 2). The GGAG haplotype (with a single AG nucleotide substitution at position -6) was found to be associated with a significant risk for SSS in comparison to the common haplotype GGAA. In addition, compared to the control group, the SSS group had a higher frequency of the G allele and a lower frequency of AA genotype for the G-6A polymorphism. Taken together, these results suggest that G-6A polymorphism is a locus significantly associated with non-familial SSS.

The frequency of the G allele in our control Taiwan Chinese population was 8.8%, which is lower than that reported in the white European population (49.6%) [30]. The frequency of the AGT G-6A AA genotype in our control population was 61.9%, which is higher than that reported in the white European population (28.6%) [30]. Recently, a rare polymorphic site in *MYH6* has been reported to be associated with high risk of non-familial SSS [6]. Apparently, non-familial SSS is one of the complex diseases associated with multiple susceptibility loci [7] such as that different ethnic population would appear the distinguished association pattern between genomic polymorphism and incidence of this syndrome.

In the present study, the luciferase activity assay demonstrated that the AGT promoter with nucleotide G at -6 had a lower transcriptional activity in comparison to the promoter with nucleotide A at -6 (Figure 4). Results obtained with a competitive binding assay on 2 different lengths of the oligonucleotide containing the specific nucleotide A or G at the -6 position of the AGT promoter indicated that a polymorphic site at the -6 position of the AGT promoter, regardless of having a length spanning from 23 to 33 nucleotides, would affect the binding affinity of the specific nuclear complex that modulates basal transcription of AGT gene expression. The EMSA result also indicates a stronger binding affinity to nuclear protein extracts for the oligonucleotide containing a nucleotide substitution for A to G at position -6. Consistent with a previous study by Inoue et al. [31], the present study strongly suggested that G-6A polymorphism involved in modulation of the AGT promoter activity is responsible for the RAS-induced fibrosis found in non-familial SSS.

In the present study, we found that the G-6A polymorphism modulates the AGT promoter activity and significantly increases the susceptibility for non-familial SSS. Based on the luciferase assay, the AGT promoter containing G at -6 showed a lower transcriptional activity than the AGT promoter containing A at -6. Accordingly, the AA genotype was determined to be the genotype with the highest transcription activity, the heterozygous AG genotype has an intermediate transcriptional activity, and the GG genotype has the lowest transcription activity. The effect of the heterozygous AG genotype on modulating AGT promoter activity is considered to be intermediate among the 3 genotypes. Therefore, the effect on the susceptibility for non-familial SSS is less with the AG genotype than with the GG genotype. In our study population, all subjects with the GG genotype of AGT G-6A polymorphism presented with non-familial SSS. However, some of the AG genotype subjects were free of bradycardia with non-familial SSS. This may be due to the intermediate modulating effect on the AGT promoter in the heterozygous AG genotype. In addition, non-familial SSS is considered as a complex disease associated with multiple susceptibility loci [6], [7]. Some subjects with AG genotype did not have the phenotype of non-familial SSS. This may be due to the complex susceptibility for SSS being determined by multiple susceptibility loci including the G-6A polymorphism and other unknown loci; this hypothesis should be investigated, along with the influence of environment factors.

A recent report by Holm H et al. provided the genome-wide association data associated with SSS [6]. Based on their results, G-6A as a polymorphic site for non-familial SSS found in this study was not included. This might be due to the complex susceptibility of SSS determined by multiple susceptibility loci, included ethnic population, and age differences. In our study, for seventy-year-old Taiwanese patients (n = 113) and controls (n = 125) the AGT promoter polymorphism G-6A was found to be highly associated with non-familial SSS (Table 2 and 3). A variant G-6A in AGT promoter appears to be a novel determinant associated with non-familial SSS in aged Taiwanese patients. It will be of interest to investigate the disturbance of RAS in the aged patients characterized with non-familial SSS in the future.

### Effects of G-6A polymorphism on heart Rate and PR interval

In addition to affecting non-familial SSS patients (Tables 2 and 3), AGT G-6A polymorphism clearly has substantial effect on sinus node function in control patients without non-familial SSS (Table 4). Since the patients with homozygous GG genotype examined in this study all appeared non-familial SSS (Table 3), heterozygous AG control patients without SSS was compared to homozygous AA control patients for evaluating the effect of G-6A polymorphism on the sinus rate in a population without non-familial SSS. In this study, the heterozygous AG patients with G allele at -6 position significantly have a 6.2 beats/min lower heart rate and a 10.8 ms longer PR interval than the AA patients based on the resting ECG examination (Table 4). In addition to the results of the resting ECG, the Holter ECG results also showed a significant trend of a lower average heart rate in the AG genotype than in the AA genotype consistent with the results of heart rate comparison in the resting ECG of the control group. These results support the findings that the AGT G-6A polymorphism has effects on sinus node function in the control subjects. In contrast to these findings for the G-6A polymorphism on heart rate and PR interval, we did not find an effect on heart rate and PR interval due to the Cx 40 -44/+71 polymorphism. These results verified the functional association of AGT G-6A polymorphism to sinus node function and rhythmic conduction in control and non-familial SSS patients.

### Electrophysiologic study findings and G-6A genotypes

Based on the estimated CSRT from the electrophysiologic study of the SSS patients, we found there were no significant differences in the CSRT values among the patients with AA, AG and GG genotypes. The results indicate that the electrophysiologic criteria for supporting SSS diagnosis were similar among the patients with AA, AG and GG genotypes, but do not indicate that the sinus node functions based on the estimated CSRT values were similar among the AA, AG and GG genotypes. In the present study, the electrophysiologic study was used to assist the diagnosis of non-familial SSS and was only performed in the SSS group. The effects of the different genotypes on the susceptibility for SSS were demonstrated by the increased incidence of genotypes with the G allele (AG and GG genotypes) in the SSS group compared with the control group. We suggest that sinus node function in AG and GG genotype should be lower than in the AA genotype with an increased CSRT in the control subject, which could be verified by obtaining the CSRT for the control group. However, we did not perform this invasive procedure in the control patients without symptoms or signs of SSS. Nevertheless, we demonstrated the results of sinus node function based on heart rate (sinus rate) measurements and showed that the AG genotype has a lower sinus rate than the AA genotype (Table 4).

### Mechanism for G-6A AGT polymorphism is associated with SSS

Cardiac pacemaker activity is regulated by different classes of ion channels [2], [5]. A recent study investigating the expression of ion channels in the human sinus node has shown the role of various ion channels (e.g., HCN, SCN5A, K^+^ channels, and Ca^++^ channels) in generating pacemaking activity [2]. Studies using transgenic knockout animals for HCN4, Ca^++^ channels, Na^+^ channels, and Cx40 demonstrated that all these ion channels, as well as gap junctions, are involved in the control of sinus node pacemaking function [2], [32]. Contrary to familial SSS, which is due to the genetic control of ion channels, non-familial SSS is believed to be closely associated with cardiac fibrosis occurring during the aging process, and during which ion channels and gap junctions may be modified by RAS disorders [12], [33]. A recent study using angiotensin converting enzyme 8/8 transgenic mice with local overexpression in cardiac tissue showed that RAS overexpression resulting in the downregulation of SCN5A and gap junction proteins leads to low-voltage electrical activity and conduction delays in the heart [34]. It appears that cardiac-specific RAS dysregulation causing changes in SCN5A and gap junction protein levels may be associated with non-familial SSS. In addition, the RAS has been reported to regulate potassium channels via the AT1 receptor in atrial myocytes derived from guinea pig hearts [32] and acts as a modulator of L-type calcium channels by activating protein kinase C via the AT1 receptor [34], [35]. These studies validate the novel action of RAS in modulating ion channels and gap junctions via the AT1 receptor, which results in a dysfunction of the pacemaking activity in the fibrotic heart tissue.

It has been reported that overexpression of the AT1 receptor causes sinus bradycardia [14]. Upregulation of AT1 receptors was observed in AGT knockout mice. These studies indicate that there is a feedback mechanism for the RAS and AT1 receptors in cardiovascular homeostasis [17]. The present study consistently shows that the SSS group, which has a higher frequency of G at position -6, has a lower AGT transcriptional activity than the control group. This suggests a unique feedback mechanism for the modulation of RAS expression, which may result in sinus node fibrosis and in the downregulation of ion channels and gap junction proteins in the heart. To our knowledge, this is the first study to demonstrate the association between G-6A AGT promoter polymorphisms and non-familial SSS, and provides functional results to clarify the underlying mechanism of non-familial SSS.

### Study limitations

Based on our study results, we found that the G-6A polymorphism is associated with non-familial SSS, and tried to utilize functional studies, including EMSA and luciferase assay, to explain the underlying mechanism of the candidate locus that affects the sinus node. However, the possible feedback mechanism of the RAS system that affects the development of non-familial SSS via modulation of AGT gene expression by G-6A polymorphism still needs additional in vivo studies for further clarification. Another limitation of the study is that the study population is small. Our results should be confirmed in a larger scale study.

### Conclusions

Patients with SSS have a lower frequency of the AGT G-6A AA genotype and a higher G allele frequency, suggesting a possible role for the AGT G-6A promoter polymorphism in determining the risk of SSS. Results obtained with the EMSA assay suggested that nucleotide substitution in AGT polymorphic site -6 would affect the promoter binding affinity for the specific nuclear complex and transcription activity. Taken together, the data reported in this study provide biological insight for the possible mechanism of non-familial SSS. The results suggest that AGT promoter G-6A polymorphisms act as modulators of the transcription activity of RAS molecules, thereby contributing to non-familial SSS.

## Supporting Information

Figure S1 **AGT A-20C genotyping by direct sequencing.** The arrows indicate the polymorphic sites of AA, AC and CC genotypes.
(TIF)

Figure S2 **AGT G-152A genotyping by direct sequencing.** The arrows indicate the polymorphic sites of GG and AG genotypes.
(TIF)

Figure S3 **AGT G-217A genotyping by direct sequencing.** The arrows indicate the polymorphic sites of GG, AA and AG genotypes.
(TIF)

Figure S4 **Cx40 -44/+71 polymorphism genotyping by direct sequencing.** The arrows indicate the polymorphic sites of the different genotypes.
(TIF)
