# Supplementary figures and images for: Promoter Polymorphism G-6A, which Modulates Angiotensinogen Gene Expression, Is Associated with Non-Familial Sick Sinus Syndrome
Source: PLoS One. 2012 Jan 5;7(1):e29951. doi: 10.1371/journal.pone.0029951 (PMC3252346; doi:10.1371/journal.pone.0029951)

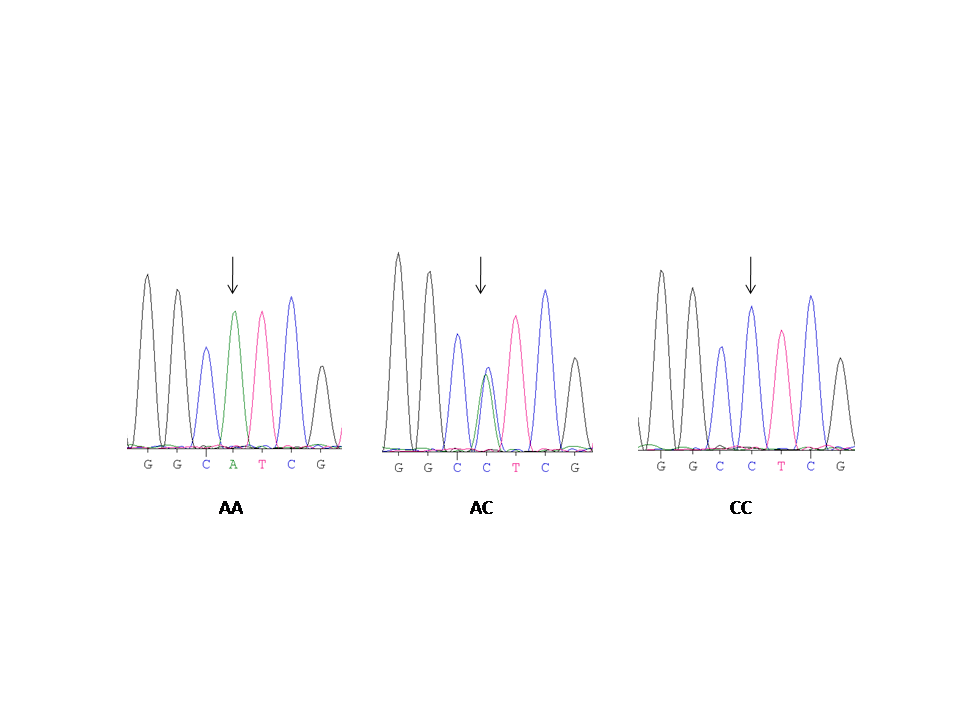

Supplement: Figure S1 — AGT A-20C genotyping by direct sequencing. The arrows indicate the polymorphic sites of AA, AC and CC genotypes. (TIF) [file pone.0029951.s001.tif]

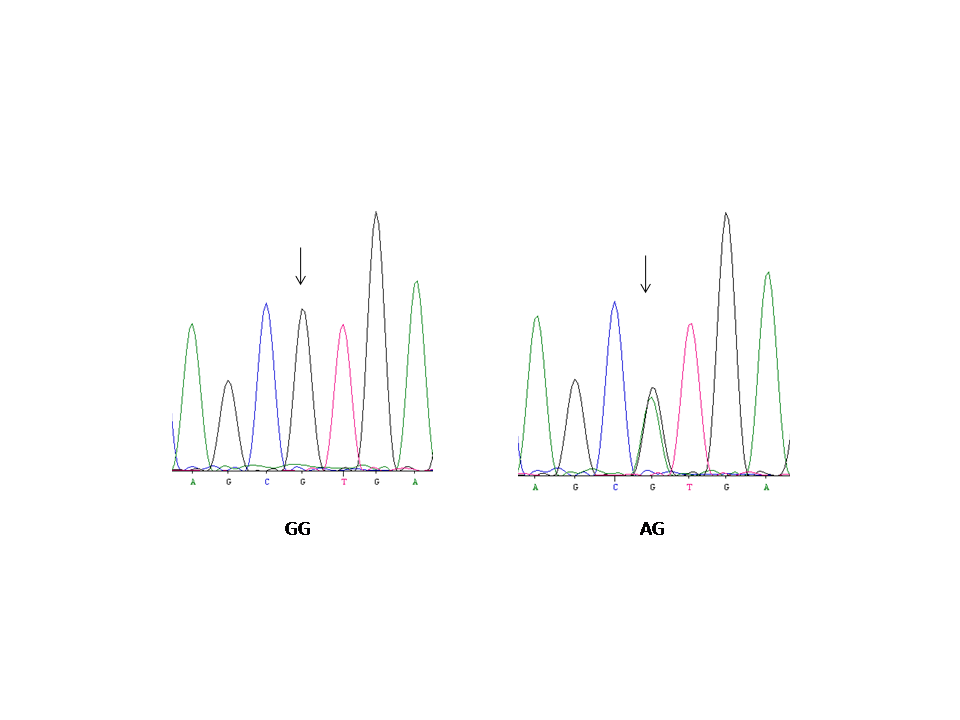

Supplement: Figure S2 — AGT G-152A genotyping by direct sequencing. The arrows indicate the polymorphic sites of GG and AG genotypes. (TIF) [file pone.0029951.s002.tif]

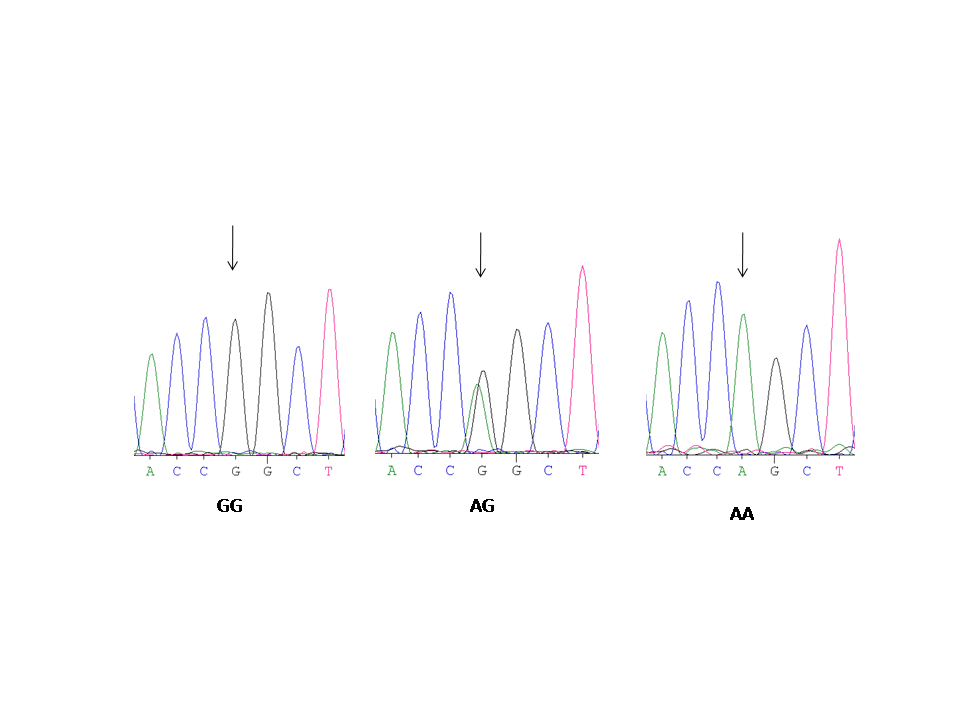

Supplement: Figure S3 — AGT G-217A genotyping by direct sequencing. The arrows indicate the polymorphic sites of GG, AA and AG genotypes. (TIF) [file pone.0029951.s003.tif]

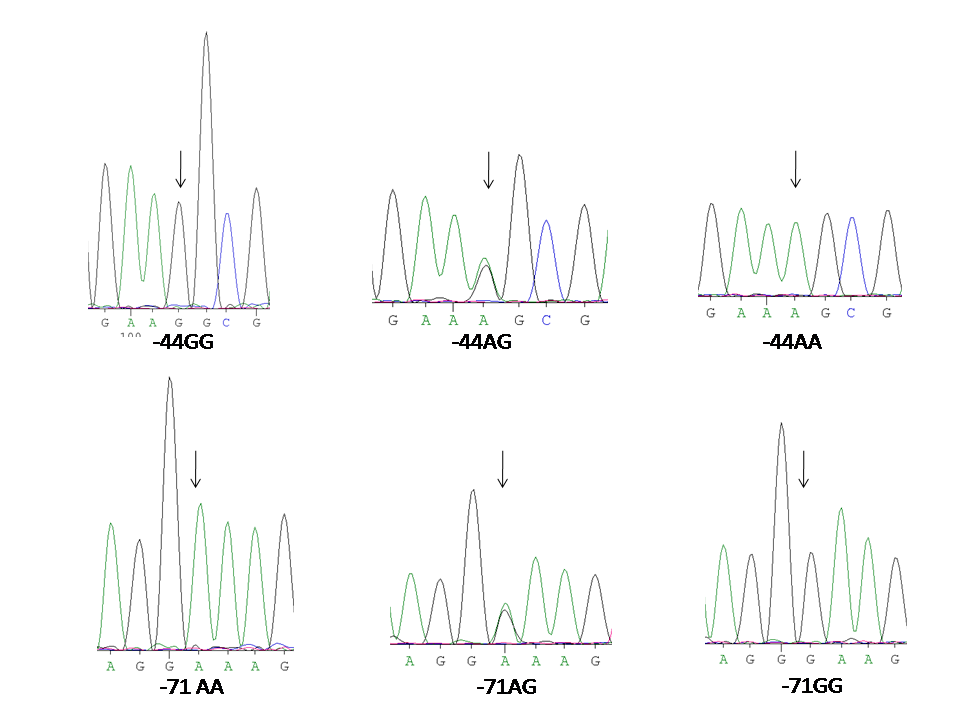

Supplement: Figure S4 — Cx40 -44/+71 polymorphism genotyping by direct sequencing. The arrows indicate the polymorphic sites of the different genotypes. (TIF) [file pone.0029951.s004.tif]
